# Lineage specific histories of *Mycobacterium tuberculosis* dispersal in Africa and Eurasia

**DOI:** 10.1111/mec.15120

**Published:** 2019-07-09

**Authors:** Mary B. O'Neill, Abigail Shockey, Alex Zarley, William Aylward, Vegard Eldholm, Andrew Kitchen, Caitlin S. Pepperell

**Affiliations:** ^1^ Laboratory of Genetics University of Wisconsin‐Madison Madison WI USA; ^2^ Department of Medical Microbiology and Immunology University of Wisconsin‐Madison Madison WI USA; ^3^ Department of Geography University of Wisconsin‐Madison Madison WI USA; ^4^ Department of Classical and Ancient Near Eastern Studies University of Wisconsin‐Madison Madison WI USA; ^5^ Infection Control and Environmental Health Norwegian Institute of Public Health Oslo Norway; ^6^ Department of Anthropology University of Iowa Iowa City IA USA; ^7^ Department of Medicine University of Wisconsin‐Madison Madison WI USA; ^8^ Present address: Unit of Human Evolutionary Genetics Institut Pasteur Paris France

**Keywords:** bacteria, landscape genetics, molecular evolution, phylogeography, population dynamics, population genetics ‐ empirical

## Abstract

*Mycobacterium tuberculosis* (*M.tb*) is a globally distributed, obligate pathogen of humans that can be divided into seven clearly defined lineages. An emerging consensus places the origin and global dispersal of *M.tb* within the past 6,000 years: identifying how the ancestral clone of *M.tb* spread and differentiated within this timeframe is important for identifying the ecological drivers of the current pandemic. We used Bayesian phylogeographic inference to reconstruct the migratory history of *M.tb* in Africa and Eurasia and to investigate lineage specific patterns of spread from a geographically diverse sample of 552 *M.tb* genomes. Applying evolutionary rates inferred with ancient *M.tb* genome calibration, we estimated the timing of major events in the migratory history of the pathogen. Inferred timings contextualize *M.tb* dispersal within historical phenomena that altered patterns of connectivity throughout Africa and Eurasia: trans‐Indian Ocean trade in spices and other goods, the Silk Road and its predecessors, the expansion of the Roman Empire, and the European Age of Exploration. We found that Eastern Africa and Southeast Asia have been critical in the dispersal of *M.tb*. Our results further reveal that *M.tb* populations have grown through range expansion, as well as in situ, and delineate the independent evolutionary trajectories of bacterial subpopulations underlying the current pandemic.

## 1 INTRODUCTION

The history of tuberculosis (TB) has been rewritten several times as genetic data accumulate from its causative agent, *Mycobacterium tuberculosis* (*M.tb*). In the nascent genomic era, these data refuted the long‐held hypothesis that human‐adapted *M.tb* emerged from an animal adapted genetic background represented among extant bacteria by *Mycobacterium bovis*, another member of the *Mycobacterium tuberculosis* complex (MTBC) (Brosch et al., 2002). Genetic data from bacteria infecting multiple species of hosts revealed that currently known nonprimate‐adapted strains form a nested clade within the diversity of extant *M.tb* (Behr et al., 1999; Brosch et al., 2002; Hershberg et al., 2008).

*Mycobacterium tuberculosis* can be classified into seven well differentiated lineages, which differ in their geographic distribution and association with human subpopulations (Gagneux et al., 2006; Hirsh, Tsolaki, DeRiemer, Feldman, & Small, 2004). This observation led some to hypothesize that *M.tb* diversity has been shaped by human migrations out of Africa, and that the most recent common ancestor (MRCA) of extant *M.tb* emerged in Africa approximately 73,000 years ago, coincident with estimated waves of human migration (Comas et al., 2013). According to this hypothesis, patterns of diversity observable in extant populations of *M.tb* are due to dispersal of the pathogen by these ancient human migrations. Human out of Africa migrations are a plausible means by which *M.tb* could have spread globally. However, *M.tb* evolutionary rate estimates based on a variety of calibration methods are inconsistent with the out of Africa hypothesis (Brynildsrud et al., 2018; Eldholm et al., 2016; Menardo, Duchene, Brites, & Gagneux, 2019).

When calibrated with ancient DNA, the estimates of the time to most recent common ancestor (TMRCA) for the MTBC are <6,000 years before present (Bos et al., 2014; Kay et al., 2015; Sabin et al., 2019). This is not necessarily the time period over which TB first emerged, as it is possible, particularly given the apparent absence of recombination among *M.tb* (Pepperell et al., 2013), that the global population has undergone clonal replacement events that displaced ancient diversity from the species.

*Mycobacterium tuberculosis* is an obligate pathogen of humans with a global geographic range. The finding of a recent origin for the extant *M.tb* population raises the question of how the organism could have spread within this timeframe to occupy its current distribution. *M.tb* populations in the Americas show the impacts of European colonial movements as well as recent immigration (Brynildsrud et al., 2018; Pepperell et al., 2011); the role of other historical phenomena in driving TB dispersal is not well understood. Here, we sought to reconstruct the migratory history of *M.tb* populations in Africa and Eurasia within the framework of a recent origin (<6,000 years before present) and evolutionary rates derived from ancient DNA data (Bos et al., 2014; Kay et al., 2015). We discovered lineage‐specific patterns of migration and a complex relationship between *M.tb* effective population growth and migration. Our results connect *M.tb* migration to major historical events in human history that altered patterns of connectivity in Africa and Eurasia. These findings provide context for a recent evolutionary origin of the MRCA of *M.tb* (Bos et al., 2014; Kay et al., 2015; Pepperell et al., 2013; Sabin et al., 2019), which represents yet another paradigm shift in our understanding of the history and origin of this successful pathogen.

## 2 MATERIALS AND METHODS

### 2.1 Lineage frequencies

The SITVIT WEB database (Demay et al., 2012), which is an open access *M.tb* molecular markers database, was accessed on 5 September 2016. Spoligotypes were translated to lineages based on the following study (Shabbeer et al., 2012). The following conversions were also included: EAI7‐BGD2 for lineage 1 (L1), CAS for lineage 3 (L3), and LAM7‐TUR, LAM12‐Madrid1, T5, T3‐OSA, and H4 for lineage 4 (L4). Isolates containing ambiguous spoligotypes (denoted with >1 spoligotype) were inspected manually and assigned to appropriate lineages. Relative lineage frequencies of lineages 1–6 for each country containing data for >10 isolates were calculated and plotted with the rworldmap package in R (South, 2016).

### 2.2 Sample description

#### 2.2.1 Old World collection

**Figure 1:**
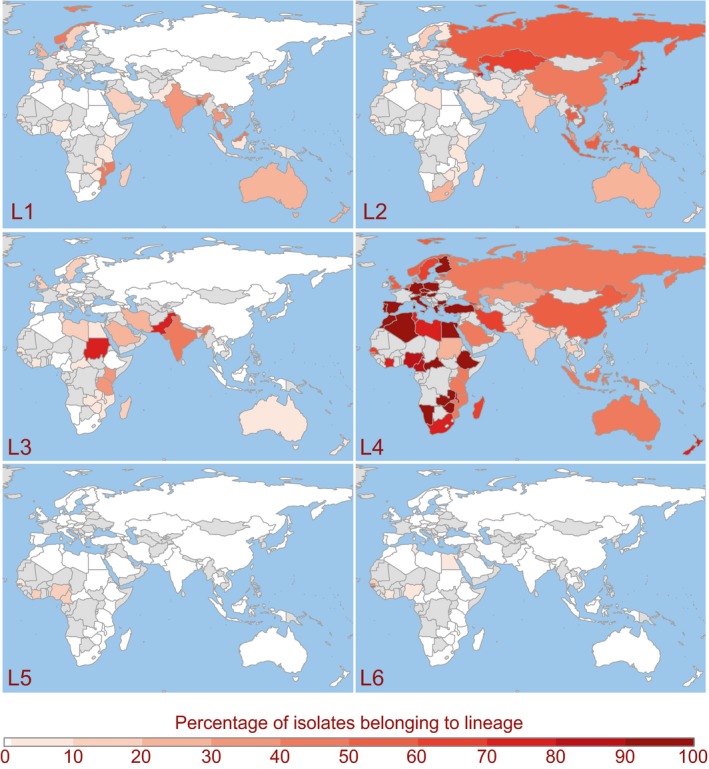
Present day distributions of country‐level relative frequencies of *Mycobacterium tuberculosis* lineages 1–6. Spoligotypes from the SITVIT WEB database (*n* = 42,358) were assigned to lineages 1–6. Countries are colored from white to dark‐red based on the percentage of isolates from the country belonging to each lineage. Unsampled countries and those with <10 isolates in the database are shown in grey. Lineage 7 (not pictured) is found exclusively in Ethiopia

We assembled/aligned publicly available whole genome sequences (WGS) of thousands of *M.tb* isolates from recently published studies and databases for which country of origin information were known and fell within regions traditionally defined as the Old World. Isolates were assembled via reference‐guided assembly (RGA) when FASTQ data were available and by multiple genome alignment (MGA) when only draft genome assemblies were accessible (see Sections 2.3 and 2.4). As we were interested in reconstructing historical migrations of the pathogen, we excluded countries where the majority of contemporary TB cases are identified in recent immigrants (Australian Government Department of Health & Ageing, 2012; Centers for Disease Control, 2015; Government of Canada, 2005; Institute of Environmental Science & Research Limited, 2014; Public Health England, 2015; White et al., 2017). Due to computational limitations (BEAST analyses), we necessarily took measures to limit our data set to <600 isolates. For countries with large numbers of available genomes, we implemented a subsampling strategy similar one previously described (Thorpe, Bayliss, Hurst, & Feil, 2017), whereby phylogenetic lineage diversity was captured thus minimizing the overrepresentation of clonal complexes (e.g., outbreaks): phylogenetic inference on all isolates available from a country was performed with Fasttree (Price, Dehal, & Arkin, 2010) and a random isolate was selected from each clade extending from *n* branches, where *n* was the desired number of isolates from the country. Numbers of isolates per country were selected based on the availability of appropriate genome sequence data as well as TB prevalence (Figure S1) (World Health Organization, 2017). All isolates belonging to lineages 5–7 were retained. As a whole, this data set reflects a “mixed” sampling scheme (Lapierre, Blin, Lambert, Achaz, & Rocha, 2016), where lineages L5–L7 are overrepresented relative to their contemporary frequencies (Figure 1). At the lineage‐specific scale, L1–L4 are phylogenetically and numerically representative subsamples. Our final Old World collection consisted of the WGS of 552 previously published *M.tb* isolates collected from 51 countries spanning 13 United Nations (UN) geoscheme subregions. Accession numbers and pertinent information about each sample can be found in Table S1.

We note that our sample necessarily contains a large number of drug‐resistant isolates as these are more commonly sequenced. We also acknowledge that the studies we draw genomes from may have been subject to other sampling biases such as under‐representation of strains from regions with limited resources.

#### 2.2.2 Northern and Central American collection

For one analysis, we included an additional 15 isolates from a previous study (Comas et al., 2015) for which the country of origin was within the Americas. Isolates were assembled via RGA (see Section2.3) and their genotypes at the 3,838,249 bp considered for all analyses of the Old World collection were extracted.

### 2.3 Reference‐guided assembly

Previously published FASTQ data were retrieved from the National Center for Biotechnology Information (NCBI) sequence read archive (SRA) (Leinonen, Sugawara, & Shumway, 2011). Low‐quality bases were trimmed using a threshold quality of 15, and reads resulting in <20 bp length were discarded using Trim Galore (http://www.bioinformatics.babraham.ac.uk/projects/trim_galore/), which is a wrapper tool around Cutadapt (Martin, 2011) and FastQC (http://www.bioinformatics.babraham.ac.uk/projects/fastqc/). Reads were mapped to H37Rv (NC_000962.3) (Cole et al., 1998) with the MEM algorithm (Li, 2013). Duplicates were removed using Picard Tools (http://picard.sourceforge.net), and local realignment was performed with GATK (DePristo et al., 2011). To ensure only high quality sequencing data were included, individual sequencing runs for which <80% of the H37Rv genome was covered by at least 20X coverage were discarded, as were runs for which <70% of the reads mapped as determined by Qualimap (García‐Alcalde et al., 2012). Pilon (Walker et al., 2014) was used to call variants with the following parameters: –variant –mindepth 10 –minmq 40 –minqual 20. Variant calls (VCFs) were converted to FASTAs with in‐house scripts that treat ambiguous calls and deletions as missing data. Transposable elements, phage elements, and repetitive families of genes (PE, PPE, and PE‐PGRS gene families) that are poorly resolved with short read sequencing were masked to missing data. Isolates with >20% missing sites were excluded from the Old World collection (Table S1).

### 2.4 Multiple genome alignment

Draft genome assemblies were aligned to H37Rv (NC_000962.3) (Cole et al., 1998) with Mugsy v1.2.3 (Angiuoli & Salzberg, 2011). Regions not present in H37Rv were removed and alignments were merged with the reference‐guided assemblies.

### 2.5 SNP alignment

Variant positions with respect to H37Rv were extracted with SNP‐sites (Page et al., 2016) resulting in 60,818 variant sites. Only sites where at least half of the isolates had confident data (i.e., nonmissing) were included in the phylogeographic models and population genetic analyses (60,787 variant sites; 3,838,249 bp). 1.7% of variant sites landed in loci associated with drug resistance (Table S5).

### 2.6 Geographic information

Geographic locations for each of the 552 samples in the Old World collection were obtained from NCBI and/or the publications in which the isolates were first described. When precise geographic information was available (e.g., city, province, etc.), coordinates were obtained from www.mapcoordinates.net. When only country level geographic information was available, the “Create Random Point” tool in ArcGIS 10.3 was used to randomly place each isolate without specific latitude and longitude inside its respective country; inhospitable areas (e.g., deserts and high mountains) and unpopulated areas from each country using 50 m data from Natural Earth (http://www.naturalearthdata.com/downloads, accessed 17 February 2016) were excluded as possible coordinates. The “precision” column of Table S1 reflects which method was used.

### 2.7 Trade route information

Data for all trade routes active throughout Europe, Africa, and Asia by 1,400 CE were compiled from the Old World Trade Routes (OWTRAD) Project (www.ciolek.com/owtrad.html, accessed 17 February 2016). For each route, both node information (trade cities, oases, and caravanserai) and arc information (the routes between nodes) were imported into ArcGIS. *M.tb* isolate locations were also imported as points and the “Generate Near Table” tool was used to assign each isolate to its nearest node in the trade network and is listed in the “NearPost” column of Table S1.

### 2.8 Maximum likelihood inference

We used RAxML v8.2.3 (Stamatakis, 2014) for maximum likelihood phylogenetic analysis of the Old World collection (all sites where at least half of isolates had nonmissing data) under the general time reversible model of nucleotide substitution with a gamma distribution to account for site‐specific rate heterogeneity. Rapid bootstrapping of the corresponding SNP alignment was performed with the ‐autoMR flag, converging after 50 replicates. Tree visualizations were created with the ggtree package in R (Yu, Smith, Zhu, Guan, & Lam, 2017).

### 2.9 Phylogeographic and demographic inference with BEAST

The Old World collection SNP alignment and individual lineage SNP alignments were analyzed using the Bayesian Markov Chain Monte Carlo coalescent method implemented in BEAST v1.8 (Drummond & Rambaut, 2007) with the BEAGLE library (Ayres et al., 2012) to facilitate rapid likelihood calculations. Analyses were performed using the general time reversible model of nucleotide substitution with a gamma distribution to account for rate heterogeneity between sites, a strict molecular clock, and both constant and Bayesian skyline plot (BSP) demographic models. Default priors were used with the exception of a lognormal distribution for population sizes. Country of origin or the UN subregion for each isolate was modelled as a discrete phylogenetic trait (Lemey, Rambaut, Drummond, & Suchard, 2009). All Markov chains were run for at least 100 million generations, sampled every 10,000 generations, and with the first 10,000,000 generations discarded as burnin; replicate runs were performed for analyses and combined to assess convergence. Estimated sample size (ESS) values of non‐nuisance parameters were >200 for all analyses. Site and substitution model choice were based on previous analyses of *M.tb* global alignments as opposed to an exhaustive comparison of models which would require unreasonable computational resources. Strict versus relaxed molecular clocks did not result in altered trends of migration at the lineage level, and comparisons between analyses using strict and relaxed clocks show strong correlation between the estimated height of nodes (e.g., *R* ^2^ > 0.97; Figure S15). Table S6 provides a summary of BEAST analyses presented and the results derived from them. Tree visualizations were created with FigTree (http://tree.bio.edu.ac.uk/software/figtree/) and the ggtree package in R (Yu et al., 2017).

### 2.10 Demographic inference from the observed site frequency spectrum (SFS)

SnpEff (Cingolani et al., 2012) was used to annotate variants with respect to H37Rv (NC_000962.3) (Cole et al., 1998) as synonymous, nonsynonymous, or intergenic. Loci at which any sequence in the population had a gap or unknown character were removed from the data set. Demographic inference with the synonymous SFS for each of the seven lineages and the entire collection was performed using ∂*a*∂*i* (Gutenkunst, Hernandez, Williamson, & Bustamante, 2009). We modeled constant population size (standard neutral model), an instantaneous expansion model, and an exponential growth model, and identified the best‐fit model and maximal likelihood parameters of the demographic model given our observed data. Our parameter estimates, *ν* and *τ*, were optimized for the instantaneous expansion and exponential growth models. Uncertainty analysis of these parameters was done using the Godambe Information Matrix (Coffman, Hsieh, Gravel, & Gutenkunst, 2016) on 100 samplings of the observed synonymous SFS with replacement and subsequent model inference.

### 2.11 Population genetic statistics

Nucleotide diversity (π) and Watterson's theta (ϴ) for various population assignments (e.g., lineage, UN subregion) were calculated with EggLib v2.1.10 (De Mita & Siol, 2012).

### 2.12 Analysis of molecular variance (AMOVA)

Analysis of molecular variances were performed using the “poppr.amova” function (a wrapper for the ade4 package [Dray & Dufour, 2007] implementation) in the poppr package in R (Kamvar, Tabima, & Grünwald, 2014). Bins were assigned via the following classification systems: UN geoscheme subregions and Level 1 (“botanical continents”) of the World geographical scheme for recording plant distributions. Isolate assignation can be found in Table S1. Genetic distances between isolates were calculated with the “dist.dna” function of the ape v4.0 package in R (Paradis, Claude, & Strimmer, 2004) from the SNP alignment of the Old World collection.

### 2.13 Mantel tests

Great circle distances between *M.tb* isolate locations were calculated with the “distVincentyEllipsoid” function in the geosphere R package (Hijmans, Williams, & Vennes, 2016). Geographic distances between isolate locations along the trade network were calculated by adding the great circle distances from the isolates to the nearest trade hubs and the shortest distance between trade hubs along the trade network; the latter was determined using an Origin‐Destination Cost Matrix and the “Solve” tool in the Network Analyst Toolbox of ArcGIS which calculates the shortest distance from each origin to every destination along the arcs in the trade network. In the event that two isolates were assigned to the same trade post, the great circle distance between the isolates was used. To calculate the geographic distance between isolates in a manner that reflects human migrations, the great circle distance between isolates and waypoints were summed. These were calculated with a custom R function (available at https://github.com/ONeillMB1/Mtb_Phylogeography) using a series of rules to define whether or not the path between isolates would have gone through a waypoint. For all three distance metrics, values were standardized. Genetic distances between isolates were calculated with the “dist.dna” function in the ape v4.0 package in R (Paradis et al., 2004) from the SNP alignment. The “mantel” function of the vegan package in R (Oksanen et al., 2017) was used to perform a Mantel test between the genetic distance matrix and each of the three geographic matrices for the Old World collection. Four of the 552 isolates were excluded from these analyses as they were from Kiribati and trade networks spanning this region were not compiled.

### 2.14 Relationship between genetic diversity and geographic distance from Addis Ababa

For this analysis, we merged the genome sequences of samples from Northern and Central America with our Old World collection. These Isolates were assembled in an identical manner to those of the Old World collection and masked at sites where less than half of the Old World collection had confident data (3,838,249 bp). For each UN subregion, the mean latitude and longitude coordinates for all *M.tb* isolates within the region were calculated. The great circle distances from these average estimates for regions to Addis Ababa were then calculated, using waypoints for between‐continent distance estimates to make them more reflective of presumed human migration patterns (Ramachandran et al., 2005). Cairo was used as a waypoint for Eastern Europe, Central Asia, Western Asia, Southern Asia, Eastern Asia, and South Eastern Asia; Cairo and Istanbul were used as waypoints for Western Europe and Southern Europe; Cairo, Anadyr, and Prince Rupert were used as waypoints for Northern and Central America. The distance between each region and Addis Ababa were the sum of the great circle distances between the two points (the average coordinates for the UN subregion and Addis Ababa) and the waypoint(s) in the path connecting them, plus the great circle distance(s) between waypoints if two were used. Treating each UN subregion as a population, the relationship between genetic diversity (assessed with π) and geographic distance from Addis Ababa were explored with linear regression for both the entire Old World collection and individual lineages in R (R Development Core Team, 2014). Code is available at https://github.com/ONeillMB1/Mtb_Phylogeography.

### 2.15 Migration rate inference

Migration rates through time were inferred from the Bayesian maximum clade credibility (MCC) trees for the entire Old World collection of *M.tb* isolates (*n = *552). Individual lineages that contain isolates from multiple UN subregions (i.e., L1: *n = *89, L2: *n = *181, L3: *n = 65*, and L4: *n = *143) were extracted and plotted separately. Only nodes with posterior probabilities greater than or equal to 80% were considered. A migration event was classified as a change in the most probable reconstructed ancestral geographic region from a parent to child node. Median heights of the parent and child nodes were treated as a range of time that the migration event could have occurred. The rate of migration through time for each lineage or the Old World collection was inferred by summing the number of migration events occurring across every year of the time‐scaled phylogeny, divided by the total number of branches in existence during each year of the time‐scaled phylogeny (both those displaying a migration event and those that do not). Code for these analyses is available at https://github.com/ONeillMB1/Mtb_Phylogeography.

Additionally, relative migration rates between UN subregions were derived from the BEAST analyses of phylogeography. The Bayesian stochastic search variable selection method (BSSVS) for identifying the most parsimonious migration matrix implemented in BEAST as part of the discrete phylogeographic migration model (Lemey et al., 2009) allowed us to use Bayes factors (BF) to identify the migration rates with the greatest posterior support and provide posterior estimates for their relative rates. Strongly supported relative rates (BF >5) and connectivity among subregions were visualized with Cytoscape v3.2.0 (Shannon et al., 2003) and superimposed onto a map generated with the “rworldmap” package in R (South, 2016).

### 2.16 Effect of selection on estimates of migration

We performed demographic forward‐in‐time simulations using the SFS_CODE package (Hernandez, 2008), which allows for demographic models with arbitrarily complex migration and selection regimes. Our simulations were performed under a simple two population model or with a more complex three population model. In all simulations, *N*_e_ for each population was 1,000, θ was 0.001 (O'Neill, Mortimer, & Pepperell, 2015), and migration between each pair of populations was symmetrical. As there is substantial evidence for little to no recombination in the *M.tb* genome, our simulations were performed without recombination.

The two population simulations were performed under three scenarios: (a) no migration between populations after initial divergence; (b) constant migration after divergence (per generation *M* = 0.5) without selection; and (c) constant migration (*M* = 0.5) with purifying selection (25% of alleles of each population have a population selection coefficient of −1.0, and the rest are neutral) after divergence.

The three population simulations were performed under five scenarios: (a) no migration between populations after simultaneous divergence of the three populations; (b) constant, symmetrical migration after divergence (per generation *M* = 0.5 for all population pairs) without selection; (c) constant, symmetrical migration (*M* = 0.5) with purifying selection (25% of alleles in all populations have a population selection coefficient of −1.0, and the rest are neutral); (d) constant, asymmetrical migration after divergence (*M* = 0.5 for migration between pop0 and pop1, *M* = 5.0 for migration between pop1 and pop2, and *M* = 0 for migration between pop0 and pop2) without selection; and (e) constant, asymmetrical migration after divergence (*M* = 0.5 between pop0 and pop1, *M* = 5.0 between pop1 and pop2, and *M* = 0 between pop0 and pop2) with purifying selection (25% of alleles in all populations have a population selection coefficient of −1.0, and the rest are neutral).

For all simulations, 25 samples were taken from each population, and sequences of 100,000 bases were generated. Twenty simulations were performed under each scenario for both the two population (60 simulations) and three population (100 simulations) models. Each sequence alignment was subsequently subjected to migration analysis in ∂*a*∂*i* (Gutenkunst et al., 2009, see Appendix S2) and BEAST v1.8.4 (Drummond & Rambaut, 2007). For each Bayesian coalescent analysis, the HKY + G substitution model, a constant population model, and a strict molecular clock model were used. A discrete symmetrical migration model (Lemey et al., 2009) was used to determine migration rates, and BSSVS (Lemey et al., 2009) was used to estimate BF support for migration rates in the three population simulations. All Markov chains were run for 10 million generations or until convergence, with samples taken every 10,000 steps, and 10% discarded as burnin. The package SpreaD3 v0.96 (Bielejec, Baele, Rodrigo, Suchard, & Lemey, 2016) was used to calculate BF support for migration rates.

## 3 RESULTS

### 3.1 Genetic and geographic structures of global *M.tb* populations

In order to establish the contemporary geographic distributions of *M.tb* lineages, we translated the spoligotypes (a molecular marker) reported for 42,358 *M.tb* isolates to their corresponding lineage designations (Figure 1). We computed relative lineage frequencies within each country based on the isolates reported in a large global database of *M.tb* molecular typing data. L1 is predominant in regions bordering the Indian Ocean, extending from Eastern Africa to Melanesia. Lineage 2 (L2) is broadly distributed, with a predominance in Eastern Eurasia and South East Asia. The geographic distribution of L3 is similar to L1 in that it is frequent among isolates reported for countries that ring the Indian Ocean, but not those extending into Southeastern Asia, and it is more frequently reported in Northern Africa and a across a broader distribution of countries in Southern Asia. L4 is strikingly well dispersed, with a predominance throughout Africa and Europe and the entire region bordering the Mediterranean. Lineages 5 (L5) and 6 (L6) are reported at low frequencies in Western and Northern Africa. Lineage 7 (L7), as previously described (Blouin et al., 2012; Comas et al., 2015; Firdessa et al., 2013), is limited to Ethiopia. We note that L1–L4 are also reported in Northern Europe, where the majority of *M.tb* cases are due to recent immigration.

We compiled a diverse collection of *M.tb* genomes for phylogenetic and population genetic inference of the demographic and migratory history of the extant *M.tb* population (see Materials and Methods). Our data set consisted of whole‐genome sequences (WGS) from 552 *M.tb* isolates collected from 51 countries (spanning 13 UN geoscheme subregions), which we refer to as the Old World collection (Figure S1, Table S1). We included sites in the alignment where at least half of these isolates had confident data (60,787 variant sites; 3,838,249 bp) for subsequent analyses, unless otherwise noted.

The inferred maximum likelihood phylogeny reveals the well described *M.tb* lineage structure, and some associations are evident between lineages and geographic regions (defined here by the UN geoscheme) (Figure S2). The phylogeny has an unbalanced shape, with long internal branches that define the lineages and feathery tips, suggestive of recent population expansion.

Genetic diversity, as measured by the numbers of segregating sites and pairwise differences (Watterson's ϴ and π), varied among lineages (Table 1, Figure S3). L1 and L4 group together and have the highest diversity; L2, L3, L5, and L6 have similar levels of diversity and form the middle grouping; L7 has the lowest diversity. We used an analysis of molecular variance (AMOVA) to delineate the effects of population subdivision on *M.tb* diversity (Table 1). The Old World collection was highly structured among UN subregions (21% of variation attributable to between‐region comparisons), whereas this structure was less apparent when regions were defined by the botanical contents outlined by the World geographic scheme for recording plant distributions (14%). This trend was reproducible when the alignment was randomly downsampled at the level of the UN region (see Materials and Methods, Figure S3). This is consistent with *M.tb's* niche as an obligate human pathogen, with bacterial population structure directly shaped by that of its host population (i.e., reflected in UN subregions) rather than climatic and other environmental features (reflected in botanical continent definitions). We obtained similar results when the lineages were considered separately (L5–L7 not analyzed due to sample sizes), except for L4, which had little evidence of population structure (4% variation among UN subregions, 2% among botanical continents).

**Table 1:** Genetic diversity of Old World *M.tb* across lineages 1–7

	*MTB*C	L1	L4	L2	L3	L5	L6	L7
Sample								
*n*	552	89	143	181	65	15	31	28
Diversity								
ϴ	2.13E‐03	7.56E‐04	7.80E‐04	4.49E‐04	3.88E‐04	1.72E‐04	3.04E‐04	7.99E‐05
Π	2.80E‐04	1.92E‐04	1.54E‐04	7.46E‐05	9.16E‐05	8.77E‐05	1.41E‐04	4.52E‐05
Demographic inference								
N/Nanc	91 ± 4	71 ± 5	55 ± 22	112 ± 102	148 ± 2	504 ± 111	50 ± 5	17 ± 4
Generations (Nanc)	0.16 ± 0.01	0.80 ± 0.06	0.65 ± 0.35	0.41 ± 0.94	3.54 ± 0.04	3.94 ± 0.73	1.10 ± 0.09	2.45 ± 0.89
LL expansion	−1,788.4	−424.2	−492.8	−467.1	−108.2	−42.4	−151.9	−64.5
LL neutral	−10,549.2	−3,246.6	−3,474.6	−2,378.9	−1,717.0	−520.7	−912.3	−159.4
*p*‐value	0.00	0.00	0.00	0.00	0.00	0.00	0.00	0.00
Structure UN subregions								
Var. between	21	19	4	20	16	NA	NA	NA
Var. within	79	81	96	80	84	NA	NA	NA
*p*‐value	<0.001	<0.001	0.002	<0.001	0.004	NA	NA	NA
Structure botanical continents								
Var. between	14	5	2	9	13	NA	NA	NA
Var. within	86	95	98	91	87	NA	NA	NA
*p*‐value	<0.001	0.02	0.05	<0.001	0.002	NA	NA	NA
TMRCA								
Median	−2,898	−360	77	−20	520	784	100	1,311
Lower	−4,032	−906	−368	−488	177	502	−339	1,152
Upper	−2,172	−10	362	279	739	964	382	1,413
Geographic origin								
1st region	W Africa	S Asia	E Africa	SE Asia	S Asia	W Africa	W Africa	E Africa
Probability	54.2%	75.6%	98.9%	81.0%	63.5%	99.9%	99.8%	99.8%
2nd region	E Africa	E Africa	E Europe	E Asia	E Africa	E Africa	E Africa	S Africa
Probability	37.5%	24.1%	0.7%	9.2%	36.2%	0.1%	0.2%	0.0%

Note

TMRCA estimates reflect scaling of results to evolutionary rates calibrated from ancient DNA (median 5.00 × 10^−8^ substitutions/ site/year [Kay et al., 2015]) and are written as calendar years. To account for uncertainty in this rate estimate, our lower and upper TMRCA estimates reflect scaling of our results with the low and high bounds of the 95% highest posterior density estimates of the rate reported from ancient DNA analysis (i.e., 4.06 × 10^−8^ and 5.87 × 10^−8^, respectively).

Abbreviations: *MTB*C*, Mycobacterium tuberculosis* complex; NA, not applicable; TMRCA, time to most recent common ancestor.

### 3.2 Distinct demographic histories of the *M.tb* lineages

**Figure 2:**
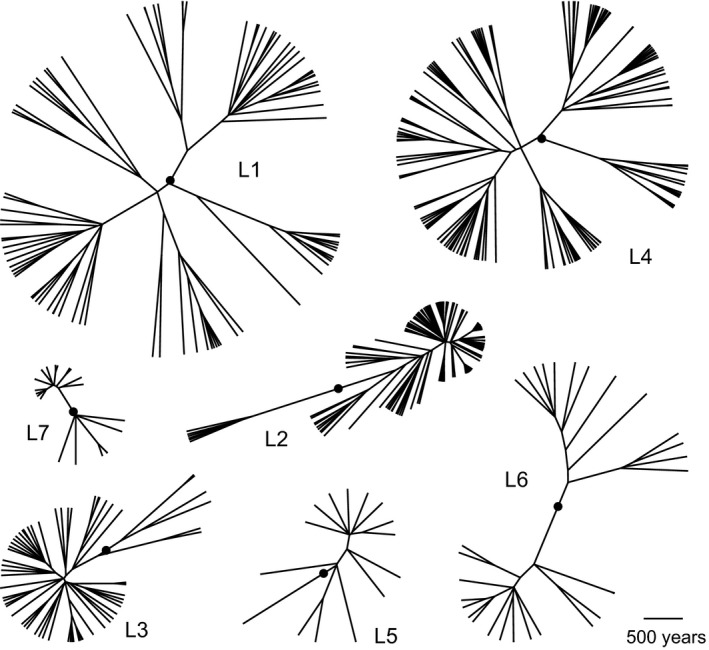
Maximum clade credibility phylogenies of *Mycobacterium tuberculosis* lineages 1–6. Bayesian analyses were performed on each lineage alignment with the general time reversible model of nucleotide substitution with a gamma distribution to account for rate heterogeneity between sites, a strict molecular clock, and Bayesian skyline plot demographic models. The most recent common ancestor (MRCA) of each lineage is indicated with a black circle; the MRCA of individual lineage phylogenies were informed by the phylogeny of the entire Old World collection, which was dated using a substitution rate of 5 × 10^−8^ substitutions/site/year (Kay et al., 2015)

**Figure 3:**
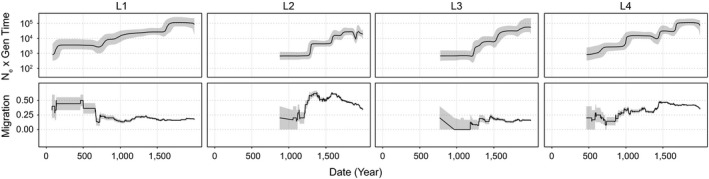
Patterns of effective population size and migration through time of *Mycobacterium tuberculosis* lineages 1–4. Bayesian skyline plots (top panels) show inferred changes in effective population size (*N*_e_) through time deduced from lineage specific analyses. Black lines denote median *N*_e_ and grey shading the 95% highest posterior density. Estimated migration through time for each lineage is shown in the bottom panels (see Section 2.15). Grey shading depicts the rates inferred after the addition or subtraction of a single migration event, and demonstrate the uncertainty of rate estimates, particularly from the early history of each lineage where branches are coalescing towards the MRCA (data omitted where less than four branches of the phylogeny are represented). Dates are shown in years and are based on scaling the phylogeny of the Old World collection with a substitution rate of 5 × 10^−8^ substitutions/site/year (Kay et al., 2015)

Bayesian inferred trees vary among lineages (Figure 2), likely reflecting their distinct demographic histories. Branch lengths are relatively even across the phylogenies of L1 and L4, whereas L2 and L3 have a less balanced structure. The long, sparse internal branches and radiating tips of L2 and L3 phylogenies are consistent with an early history during which the effective population size remained small (and diversity was lost to drift), followed by more recent population expansion. L5 has a star‐like structure, consistent with rapid population expansion. Jointly inferred Bayesian skyline plot (BSP) reconstructions of effective population sizes over time suggest that lineages 1–6 have undergone expansion (Figure 3, top panel, Figure S4).

We used the methods implemented in ∂*a*∂*i* (Gutenkunst et al., 2009) to reconstruct the demographic histories of each *M.tb* population (i.e., lineage) from its synonymous site frequency spectrum (SFS). As demographic inference with ∂*a*∂*i* is sensitive to missing data, loci at which any sequence in the individual lineage alignments had a gap or unknown character were removed for these analyses. Consistent with the BSP analyses performed in BEAST, instantaneous expansion and exponential growth models offered an improved fit to the data in comparison with the constant population size model for each lineage and the entire Old World collection (Figure S5). The exponential growth model, while likely more realistic, did not produce replicable results when implemented in ∂*a*∂*i* as indicated by widely varying parameter estimates across runs, so we report results only for the instantaneous expansion model (Table 1).

### 3.3 Major events in *M.tbs* migratory history

**Figure 4:**
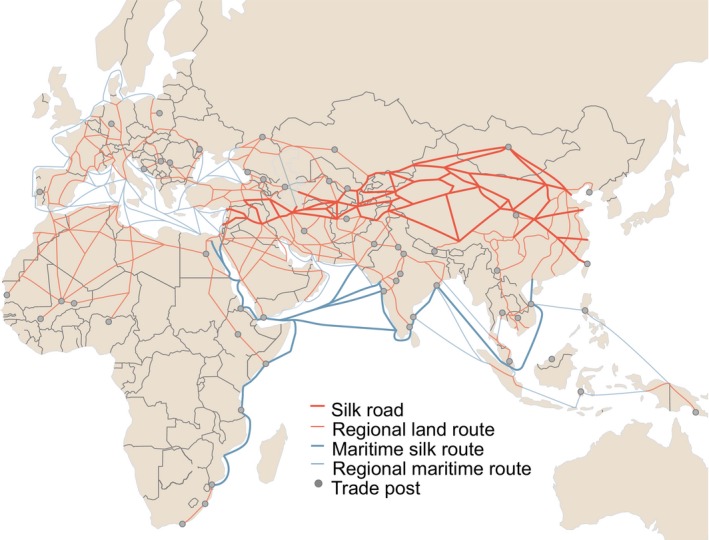
Trade routes active throughout Europe, Africa and Asia by 1,400 CE. Nodes (trade cities, oases, and caravanserai) and arcs (the routes between nodes) are from the Old World Trade Routes Project (www.ciolek.com/owtrad.html, accessed 17 February 2016) and are visualized with ArcGIS

There was evidence of isolation by distance in the global *M.tb* population, as assessed with a Mantel test of correlation between genetic and geographic distances (Figure S3). To test whether the global diversity of *M.tb* was shaped by historical human movements, we defined geographic distances using three schemes: great circle distances, great circle distances through five waypoints described in (Ramachandran et al., 2005), and distances along historical trade routes active throughout Europe, Africa and Asia by 1,400 CE (Figure 4). The waypoints described in (Ramachandran et al., 2005) are used to make distance estimates more reflective of presumed migration patterns of humans populating the world (i.e., it is generally thought that early human migrations out of Africa occurred over land rather than across large bodies of water). To allow comparisons between the schemes, values were standardized (see Materials and Methods). Values of the Mantel test statistic were highest for trade network distance (*r* = 0.19) followed by great circle distances (*r* = 0.18), while distances through the five waypoints had a lower value (*r* = 0.16, *p* = 0.0001 for all three analyses). Mantel test statistic values were lower when considering subsamples of the Old World Collection, but the relationship among the three schemes remained the same the majority of the time (Figure S3). This is in contrast with analyses of human genetic data, where adjustment of great circle distances with these waypoints results in a higher correlation between genetic and geographic distances (Ramachandran et al., 2005). Our Mantel test results therefore do not support the previously proposed hypothesis that ancient human migrations out of Africa were the primary influence on global diversity of extant *M.tb* (Comas et al., 2013).

To further investigate a potential influence of ancient human migration on *M.tb* evolution, we calculated the correlation between *M.tb* genetic diversity (π) within subregions and their average distances from Addis Ababa, a proxy for a possible origin of anatomically modern human expansion out of Africa. Contrary to what is observed for human population diversity (Ramachandran et al., 2005) and what was previously reported for *M.tb* (Comas et al., 2013), we did not observe a significant decline in *M.tb* diversity as a function of distance in our Old World collection (adjusted *R* ^2^ = –0.1, *p* = 0.88), nor when we included samples from the Americas (adjusted *R* ^2^ = 8.9 × 10^−4^, *p* = 0.34, Appendix S1, Figure S6, Table S2).

**Figure 5:**
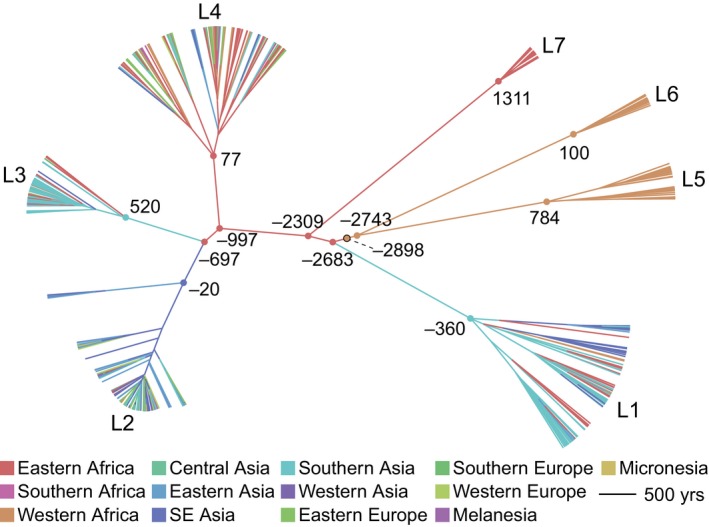
Maximum clade credibility tree of the Old World Collection. Estimated divergence dates are shown in years based on median heights and a substitution rate of 5 × 10^−8^ substitutions/site/year (Kay et al., 2015). Negative numbers refer to BCE and positive numbers refer to CE. Branches are coloured according to the inferred most probable geographic origin. Nodes corresponding to the most recent common ancestors (MRCA) of each lineage, lineage splits, and the MRCA of *Mycobacterium tuberculosis* (outlined black) are marked with circles and coloured to reflect their most probable geographic origin

We used the methods implemented in BEAST to reconstruct the migratory history of the entire Old World *M.tb* collection as well as individual lineages within it, modelling geographic origin of isolates (UN subregion or country) as a discrete trait (Figure 5, Figures [Link], [Link]). Using an evolutionary rate calibrated with 18th century *M.tb* DNA of 5 × 10^−8^ substitutions/site/year (Kay et al., 2015), which is similar to the rate inferred with data from 1,000 year old specimens (Bos et al., 2014), our estimate of the time to most recent common ancestor for extant *M.tb* is between 4,032 BCE and 2,172 BCE (Table 1; date ranges are based on the upper and lower limits of the 95% highest posterior density [HPD] for the rate reported in Kay et al. (2015) which is more conservative than the 95% HPD of our model). We infer an African origin for the MRCA (Eastern or Western subregion, Table 1, Figures 5 and S7). Shortly after emergence of the common ancestor, we infer a migration of the L1‐L2‐L3‐L4‐L7 ancestral lineage from Western to Eastern Africa (we estimate prior to 2,683 BCE), with subsequent migrations occurring out of Eastern Africa.

In our phylogeographic reconstruction, emergence of L1 follows migration from Eastern Africa to Southern Asia at some time between the third millennium and 4th century BCE (Table 1, Figures 5 and S7). L1 has an “out of India” phylogeographic pattern (Figure S8), with diverse Indian lineages interspersed throughout the phylogeny. This suggests that the current distribution of L1 around the Indian Ocean (Figure 1) arose from migrations out of India, from a pool of bacterial lineages that diversified following migration from Eastern Africa.

The phylogeographic reconstruction further indicates that following the divergence of L1, *M.tb* continued to diversify in Eastern Africa, with emergence of L7 there, followed by L4 (Table 1, Figures 5 and S7). The contemporary distribution of L4 is extremely broad (Figure 1) and in this analysis of the Old World collection we infer an East African location for the internal branches of L4. Notably, in the lineage‐specific analyses, we infer a European location for these branches (Figure S9). The difference is likely due to the fact that inference is informed by deeper, as well as descendant nodes in the Old World collection. Together, these results imply close ties between Europe and Africa during the early history of this lineage that we estimate emerged in the 1st century CE (368 BCE–362 CE, Table 1).

After the emergence of L1 and L7 from Eastern Africa, our analyses suggest that a migration occurring between 697 BCE and 520 CE established L3 in Southern Asia, with subsequent dispersal out of Southern Asia into its present distribution, which includes Eastern Africa (i.e., a back migration of L3 to Africa, Figure 1). We estimate that L2 diversified in South Eastern Asia following migration from Eastern Africa at some point between 697 BCE and 20 BCE (Table 1, Figures 5 and S7). Previously published analyses of L2 phylogeography also inferred a Southeast Asian origin for the lineage (Liu et al., 2018; Luo et al., 2015).

### 3.4 Lineage and region specific patterns of migration

Our phylogeographic reconstruction indicated that temporal trends in migration varied among lineages (Figure 3, bottom panel). We infer that L1 was characterized by high levels of migration until approximately the 7th century CE, when the rate of migration decreased abruptly and remained stable thereafter. L3, by contrast, exhibited consistently low rates of migration. L2 and L4 had more variable trends in migration, as each underwent punctuated increases in migration rate. Temporal trends in growth and migration are congruent for L2 and L4, with increases in migration rate preceding effective population expansions; this is not the case for L1 and L3. Taken together, these results suggest that L1 and L3 populations (as well as L5 and L6, Figure S4b) grew in situ, whereas range expansion may have contributed to the growth of L2 and L4.

**Figure 6:**
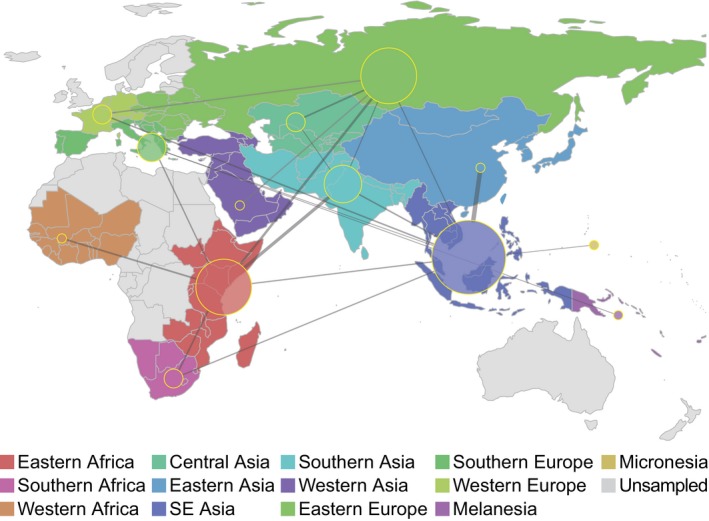
Connectivity of UN subregions during dispersal of *Mycobacterium tuberculosis*. The Bayesian stochastic search variable selection method was used to identify and quantify migrations with strong support in discrete phylogeographic analysis of the Old World collection. Node sizes reflect the number of significant migrations emanating from the region observed in the phylogeny, whereas the thickness of lines connecting regions reflects the estimated relative rate between regions. Inferred rates are symmetric and do not indicate directionality

We employed the Bayesian stochastic search variable selection method (BSSVS) in BEAST (Lemey et al., 2009) to estimate relative migration rates within the most parsimonious migration matrix. A map showing inferred patterns of connectivity among UN subregions and relative rates of *M.tb* migration with strong posterior support is shown in Figure 6. South Eastern Asia was the most connected region in our analyses, with significant rates of migration connecting it to eight other regions. Eastern Africa, Eastern Europe, and Southern Asia were also highly connected, with significant rates with six, six, and five other regions, respectively. Western Africa, Eastern Asia, and Western Asia were the least connected regions, with just one significant connection each (to Eastern Africa, South Eastern Asia, and Eastern Europe, respectively). Our sample from Western Asia is, however, limited (Table S1) and migration from this region may have consequently been underestimated. The highest rates of migration were seen between Eastern Asia and Southeastern Asia, and between Eastern Africa and Southern Asia.

Lineage specific analyses suggest that migration between Southern Asia, Eastern Africa, and South Eastern Asia has been important for the dispersal of L1, whereas South Eastern Asia and Eastern Europe have been important for L2 (Figure S12). L3 is similar to L1 in that there is evidence of relatively high rates of migration between Southern Asia and Eastern Africa. There is also evidence of migration within Africa between the eastern and southern subregions. In the analyses of migration for L4, Eastern Africa appeared highly connected with other regions.

## 4 DISCUSSION

Our reconstructions of *M.tb* dispersal throughout the Old World delineate a complex migratory history that varies substantially between bacterial lineages. Patterns of diversity among extant *M.tb* suggest that historical pathogen populations were capable of moving fluidly over vast distances. Using evolutionary rate estimates from ancient DNA calibration, we time the dispersal of *M.tb* to a historical period of exploration, trade, and increased connectivity among regions of the Old World.

Consistent with prior reports (Comas et al., 2013), we infer an origin of *M.tb* on the African continent (Table 1, Figures 5 and S7). There is a modest preference for Western Africa over Eastern Africa (54% vs. 38% inferred probability), likely due to the early branching West African lineages (i.e., *Mycobacterium africanum*, L5 and L6). Larger samples may allow more precise localization of the *M.tb* MRCA, and Northern Africa in particular is under‐studied.

We infer L1 to be the first lineage that emerged out of Africa; L1 is currently among the most frequently reported lineages in countries bordering the Indian Ocean from Eastern Africa to Melanesia (Figure 1). In our phylogeographic reconstruction, the genesis of this lineage traces to migration from Eastern Africa to Southern Asia at some point between the third millennium and 4th century BCE, with subsequent dispersal occurring out of the Indian subcontinent. Our results suggest that the early history of L1 was characterized by high levels of migration, particularly between Southern Asia and Eastern Africa, and between Southern Asia and South Eastern Asia (Figures 3 and S12). Given the geographic distribution of L1, the timing of its emergence and spread, as well as patterns of connectivity underlying its dispersal, we hypothesize that spread of this lineage was facilitated via established trans‐Indian Ocean trade routes linking Eastern Africa to Southern and South Eastern Asia (Figure 4). The interval of our timing estimate for the initial migration overlaps with the so‐called Middle Asian Interaction sphere in The Age of Integration (2600–1900 BCE), which is marked by increased cultural exchange and trade between civilizations of Egypt, Mesopotamia, the Arabian peninsula, and the Indus Valley (Coningham & Young, 2015; Parkin & Barnes, 2002; Ray, 2003; Vogt, 1996; Zarins, 1996). East‐West contact and trade across the Indian Ocean intensified in the first millennium BCE, when maritime networks expanded to include the eastern Mediterranean, the Red Sea, and the Black Sea (Boussac & Salles, 1995; Dilke, 1985; Ray & Salles, 1996; Salles, 1996). Historical data from the Roman era indicate that crews on trading ships crossing the Indian Ocean comprised fluid assemblages of individuals from diverse regions, brought together under conditions favourable for the transmission of TB (André & Filliozat, 1986; Begley & De Puma, 1991; Rauh, 2003; Wink, 2002). These ships would have been an efficient means of spreading *M.tb* among the distant regions involved in trade.

L2 may similarly have an origin in East‐West maritime trade across the Indian Ocean, as we infer it arose from a migration event from Eastern Africa to South Eastern Asia during the 1st millennium BCE. In this era, increased sophistication in ship technology allowed for longer voyages (Blench, 1996; Kent, 1979; Parkin & Barnes, 2002; Ray, 2003; Ray & Salles, 1996; Wink, 2002). L2 appears to have spread out of Southeast Asia, a highly connected region in our analyses of *M.tb* migration, and is currently found across Eastern Eurasia and throughout South Eastern Asia (Figures 1, 5, S7 and S12). Although L2 is dominant in Eastern Asia, interestingly the region did not appear to have played a prominent role in dispersal of this lineage, except in its exchanges with South Eastern Asia. A recently published study using a range of methods including Bayesian inference on a large regional sample found that the extant *M.tb* population in China traces to a limited number of introductions (Liu et al., 2018), which is consistent with our findings of relatively few exchanges of *M. tb* between Eastern Asia and other regions.

L3 appears to have had relatively low rates of migration throughout its history (Figure 3). The contemporary geographic range of L3 is also narrower, extending east from Northern Africa through Western Asia to the Indian subcontinent (Figure 1). A study of lineage prevalence in Ethiopia showed that L3 is currently concentrated in the north of the country (Comas et al., 2015), consistent with our observed north to south gradient in its distribution on the African continent. This is in opposition to L1, which was found previously to have a southern predominance in Ethiopia (Comas et al., 2015) and, in our analyses, was predominant in Southeastern Africa (Figure 1). We estimate L3 emerged in Southern Asia ca. 520 CE (177–739 CE). Pakistan harbors diverse strains belonging to L3 (Figure S10), and the Southern Asia region was highly connected with Eastern Africa in our analyses (Figure S12). Trade along the Silk Road connecting Europe and Asia was very active in the middle of the first millennium, when we estimate L3 emerged (Ball, 2016; Hansen, 2012); its distribution suggests it feasibly could have spread primarily along trading routes connecting Northeast Africa, Western Asia, and South Asia (André & Filliozat, 1986; Ball, 2016; Hansen, 2012; Sartre, 1991) (Figure 4). We speculate that this occurred via overland routes, which may have limited the migration of L3 relative to our hypothesized maritime dispersal of the other lineages.

The geographic distribution of L4 is strikingly broad (Figure 1) and it exhibits minimal population structure (Table 1). This suggests L4 dispersed efficiently and continued to mix fluidly among regions, a pattern we would expect if it was carried by an exceptionally mobile population of hosts. L4 is currently predominant among reported lineages in regions bordering the Mediterranean, and elsewhere throughout Africa and Europe (Figure 1). We estimate the MRCA of L4 emerged in the 1st century CE (range 368 BCE–362 CE), during the peak of Roman Imperial power across the entire Mediterranean world and expansionist Roman policies into Africa, Europe, and Mesopotamia (Isaac, 2004; Luttwak, 1976). The empire reached its greatest territorial extent in the early second century CE, when all of North Africa, from the Atlantic Ocean to the Red Sea, was under a single power, with trade on land and sea facilitated by networks of stone‐paved roads and protected maritime routes (Ball, 2016; Luttwak, 1976; Millar, 1993). Primary sources from Roman civilization attest to trade with China, purposeful expeditions for exploration, cartography, and trade in the Red Sea and Indian Ocean (Begley & De Puma, 1991; Butcher, 2003; Dilke, 1985; Erdkamp, 2002; Pfister & Bellinger, 1945).

We hypothesize that the broad distribution of L4 reflects rapid diffusion from the Mediterranean region along trade routes extending throughout Africa, the Middle East, and on to India, China, and South Eastern Asia. High rates of migration appear to have been maintained for this lineage over much of its evolutionary history (Figure 3); patterns of connectivity implicate Europe and Africa in its dispersal (Figure S12). The association of L4 with European migrants is well described, particularly migrants to the Americas (Brynildsrud et al., 2018; Gagneux et al., 2006; Pepperell et al., 2011). Here we note bacterial population growth preceded geographic range expansion in L4 ~ca. 15th century (Figure 3), which coincides with the onset of the “age of exploration” (Alam & Subrahmanyam, 2009) that would have provided numerous opportunities for spread of this lineage from Europeans to other populations. We also note the origin and contemporary predominance of this lineage on the African continent. Our sample of L4 isolates includes several deeply rooting African isolates, and African isolates are interspersed throughout the phylogeny (Figures 5, S7 and S9).

The migratory histories of L5, L6, and L7 are less complicated than those of lineages 1–4. Specifically, L5 and L6 are restricted to Western Africa and L7 is found only in Ethiopia (Figures 5 and S7). The reasons for the restricted distributions of these lineages are not immediately obvious: there is evidence in our analyses that other lineages migrated in and out of Western Africa, and Eastern Africa emerged as highly connected and central to the dispersal of *M.tb* (Figure 6). A potential explanation is restriction of the pathogen population to human subpopulations with distinct patterns of mobility and connectivity that did not facilitate dispersal. This is likely the case for L7, which was discovered only recently (Blouin et al., 2012), and is currently largely restricted to the highlands of northern Ethiopia (Comas et al., 2015; Firdessa et al., 2013). In the case of L6 (also known as *Mycobacterium africanum*), there is evidence suggesting infection is less likely to progress to active disease than for *M. tuberculosis* sensu* stricto* (Jong et al., 2008), which could have played a role in limiting its dispersal.

Our reconstructions of *M.tb's* migratory history suggest that patterns of migration were highly dynamic: the pathogen appears to have dispersed efficiently, in complex patterns that nonetheless preserved the distinct structure of each lineage. Some findings, notably inference of population expansion, were consistent across lineages. Growth of the global *M.tb* population has been described previously (Comas et al., 2013; Pepperell et al., 2013); our results here suggest that the pace and magnitude of expansion, and its apparent relationship to trends in migration, varied among lineages (Figure 3, Figures S4 and S12).

Our analyses suggest that the expansion of L2 was preceded by an impressive increase in its rate of migration (Figure 3), implying that growth of the pathogen population was facilitated by expansion into new niches. Our phylogeographic reconstructions implicate Russia, Central Asia, and Western Asia in the recent migratory history of L2 (Figures S11 and S12), which is consistent with a published phylogeographic analysis of L2 (Luo et al., 2015). The inferred timing of the growth and increased migration of L2 (~ca. 13th century) is close to the well documented incursion of *Yersinia pestis* from Central Asia into Europe that resulted in explosive plague epidemics (Benedictow, 2004). The experience with plague suggests that patterns of connectivity among humans and other disease vectors were shifting at this place and time, which would potentially open new niches for pathogens including *M.tb*.

We estimate that L1 underwent expansion ~ca. 17th century (Figure 3) but in this case it appears to have grown in situ, e.g., due to changing environmental conditions such as increased crowding, and/or growth of local human populations. A study of the molecular epidemiology of TB in Vietnam identified numerous recent migrations of L2 and L4 into the region, versus a stable presence of L1 (Holt et al., 2018); this is consistent with our finding of higher recent rates of migration for L2 and L4 versus L1 (Figure 3). A pattern similar to L1 has been identified previously, in the delay between dispersal of *M.tb* from European migrants to Canadian First Nations and later epidemics of TB driven by shifting disease ecology (Pepperell et al., 2011). These results demonstrate the complex relationship between *M.tb* population growth and migration, and show that under favourable conditions the pathogen can expand into novel niches or accommodate growth in an existing niche.

In a previous study, analyses of synonymous and nonsynonymous SFS have been used to delineate effects of purifying selection, linkage of sites, and population expansion on global populations of *M.tb* (Pepperell et al., 2013). Simulation studies have shown that purifying selection can affect demographic inference with BEAST and SFS‐based methods (Ewing & Jensen, 2015; Lapierre et al., 2016). Although our analyses here using ∂*a*∂*i* (Gutenkunst et al., 2009) were restricted to synonymous SFS, it is likely that inference of population size changes with this method and with BEAST were affected by purifying selection on this fully linked genome. The magnitude of inferred expansions may thus reflect both population size changes and background selection and should not be interpreted as direct reflections of historical changes in census population size. We did not detect an effect of purifying selection on inference of migration in our three population simulation analyses (Appendix S2, Figures S13 and S14), but differences in the strength of purifying selection could contribute to the lineage‐specific differences we observed in the size of inferred population expansions: i.e., genome‐wide patterns of purifying selection could differ among lineages. Previous evidence has suggested that the fitness trade‐offs of drug resistance mutations vary among lineages (Mortimer, Weber, & Pepperell, 2018), making this intriguing possibility potentially feasible.

This study has some important limitations. The phylogeographic reconstructions are clearly sensitive to sampling, since we cannot identify the roles of unsampled regions in *M.tb's* migratory history. We maximized geographic diversity in our sample but were limited by available data and some regions – notably Middle Africa, Northern Africa, and Western Asia – are absent or underrepresented in our sample (Figure S1). Defining the contributions of these undersampled regions to *M.tb's* migratory history awaits more samples and/or further method development.

De Maio, Wu, O'Reilly, and Wilson (2015) note the sensitivity of discrete trait phylogeographic inference in BEAST to sample selection, as well as overconfidence in the precision of geographic inference, and propose BASTA as an alternative (De Maio et al., 2015). BASTA is sensitive to the choice of prior and we did not have ancillary data to guide the selection of a prior for the Old World migratory history of *M.tb*, precluding its use here. We investigated ∂*a*∂*i* as an alternative tool for phylogeographic inference but it did not perform well for this application under conditions of complete linkage of sites (Appendix S3, Figures S16 and S17, Tables S3 and S4). The phylogeographic inference method implemented here relies on the assumption that sample size reflects deme size (Lemey et al., 2009; De Maio et al., 2015), and within the constraints of available data, we attempted to adjust our sample sizes according the regional prevalence of TB (see Section 2.2.1 and Figure S1). We also interrogated the relationship between regional sample size and inferred migration rate and did not observe a strong correlation (Figure S18). According to the classifications proposed by Lapierre et al. (2016), our Old World collection represents a “mixed” sampling scheme (see Section 2.2.1). Further phylogeographic method development allowing larger sample sizes, as well as larger samples from understudied but epidemiologically important regions, will lead to further refinements of the phylogeographic inferences made here. We do note that the major trends identified here are consistent with a large body of literature that includes WGS and other molecular typing modalities.

We did not attempt to estimate the rate or timescale of *M.tb* evolution, instead relying on published rates that were calibrated with ancient DNA. This is an active area of research, and newly discovered ancient *M.tb* DNA samples will likely refine inference of both the timing and locations of historical migration events, though it is critical to note that recent substitution rate estimates of *M.tb* have converged on rates around 5 × 10^−8^ substitutions per site per year (Eldholm et al., 2016). Even when substitution rate estimates can be estimated with confidence, the precision with which individual events can be dated using genetic data should not be over‐stated, as evidenced by broad 95% credible intervals for internal node date estimates (e.g., Eldholm et al., 2016). Our goal here was to reconstruct historical migration of *M.tb* throughout Eurasia and Africa and place this evolutionary history within a broad historical context; the historical phenomena that we connect with the spread of TB involved vast areas and extended over hundreds and in some cases thousands of years. Our reconstruction of the global dispersal of TB within a temporal framework provided by ancient *M.tb* DNA analysis links spread of the disease to the first ~1,500 years of the common era, a period of remarkable intensification in the connectedness among peoples of Africa, Asia and Europe (Green, 2018).

## AUTHOR CONTRIBUTION

C.S.P., M.B.O., and A.K. conceived and designed the project. M.B.O., A.K., A.S., and A.Z. performed the analyses and all authors interpreted the data. C.S.P. and M.B.O. drafted the manuscript and all authors provided critical feedback, reviewed, and edited the manuscript. All authors approved the final manuscript.

## References

[mec15120-bib-0001] Alam, M. , & Subrahmanyam, S. (2009). Indo‐Persian travels in the age of discoveries, 1400–1800. Digit. pr. Cambridge, UK: Cambridge University Press.

[mec15120-bib-0002] André, J. , & Filliozat, J. (1986). L'Inde vue de Rome: Textes latins de l'antiquité, relatifs à l'Inde. Paris, France: Les Belles Lettres.

[mec15120-bib-0003] Angiuoli, S. V. , & Salzberg, S. L. (2011). Mugsy: Fast multiple alignment of closely related whole genomes. Bioinformatics, 27, 334–342. 10.1093/bioinformatics/btq665 21148543 PMC3031037

[mec15120-bib-0004] Australian Government Department of Health and Ageing . (2012). Tuberculosis notifications in Australia, 2008 and 2009. Communicable Diseases Intelligence, 36. Retrieved from http://www.health.gov.au/internet/main/publishing.nsf/Content/cda-cdi3601c.htm#refs 10.33321/cdi.2012.36.3 23153084

[mec15120-bib-0005] Ayres, D. L. , Darling, A. , Zwickl, D. J. , Beerli, P. , Holder, M. T. , Lewis, P. O. , … Suchard, M. A. (2012). BEAGLE: An application programming interface and high‐performance computing library for statistical phylogenetics. Systems Biology, 61, 170–173. 10.1093/sysbio/syr100 PMC3243739 21963610

[mec15120-bib-0006] Ball, W. (2016). Rome in the East: The transformation of an Empire, 2nd ed. New York, NY: Routledge.

[mec15120-bib-0007] Begley, V. , & De Puma, R. D. (1991). Rome and India: The ancient sea trade. Madison, WI: University of Wisconsin Press.

[mec15120-bib-0008] Behr, M. A. , Wilson, M. A. , Gill, W. P. , Salamon, H. , Schoolnik, G. K. , Rane, S. , & Small, P. M. (1999). Comparative genomics of BCG vaccines by whole‐genome DNA microarray. Science, 284, 1520–1523. 10.1126/science.284.5419.1520 10348738

[mec15120-bib-0009] Benedictow, O. J. (2004). The Black death, 1346–1353: The complete history. Woodbridge, UK: Boydell Press.

[mec15120-bib-0010] Bielejec, F. , Baele, G. , Rodrigo, A. G. , Suchard, M. A. , & Lemey, P. (2016). Identifying predictors of time‐inhomogeneous viral evolutionary processes. Virus Evolution, 2, 023 10.1093/ve/vew023 PMC5072463 27774306

[mec15120-bib-0011] Blench, R. (1996). The ethnographic evidence for long‐distance contacts between Oceania and East Africa In Reade J. (Ed.), The Indian Ocean in antiquity (pp. 417–438). London, UK: Kegan Paul.

[mec15120-bib-0012] Blouin, Y. , Hauck, Y. , Soler, C. , Fabre, M. , Vong, R. , Dehan, C. , … Vergnaud, G. (2012). Significance of the identification in the Horn of Africa of an exceptionally deep branching *Mycobacterium tuberculosis* clade. PLoS ONE, 7, e52841 10.1371/journal.pone.0052841 23300794 PMC3531362

[mec15120-bib-0013] Bos, K. I. , Harkins, K. M. , Herbig, A. , Coscolla, M. , Weber, N. , Comas, I. , … Krause, J. (2014). Pre‐Columbian mycobacterial genomes reveal seals as a source of New World human tuberculosis. Nature, 514, 494–497. 10.1038/nature13591 25141181 PMC4550673

[mec15120-bib-0014] Boussac, M.‐F. , & Salles, J.‐F. (Eds.) (1995). Athens, Aden, Arikamedu: Essays on the interrelations between India, Arabia, and the eastern Mediterranean. New Delhi, India: Manohar, Distributed in South Asia by Foundation Books.

[mec15120-bib-0015] Brosch, R. , Gordon, S. V. , Marmiesse, M. , Brodin, P. , Buchrieser, C. , Eiglmeier, K. , … Cole, S. T. (2002). A new evolutionary scenario for the *Mycobacterium tuberculosis* complex. Proceedings of the National Academy of Sciences of the United States of America, 99, 3684–3689. 11891304 10.1073/pnas.052548299 PMC122584

[mec15120-bib-0016] Brynildsrud, O. B. , Pepperell, C. S. , Suffys, P. , Grandjean, L. , Monteserin, J. , Debech, N. , … Eldholm, V. (2018). Global expansion of *Mycobacterium tuberculosis* lineage 4 shaped by colonial migration and local adaptation. Science Advances, 4, eaat5869. 30345355 10.1126/sciadv.aat5869 PMC6192687

[mec15120-bib-0017] Butcher, K. (2003). Roman Syria and the Near East. Los Angeles, CA: J. Paul Getty Museum, Getty Publications.

[mec15120-bib-0018] Centers for Disease Control . (2015). CDC ‐ Reported tuberculosis in the United States TB (2015). Retrieved from https://www.cdc.gov/tb/statistics/reports/2015/default.htm.

[mec15120-bib-0019] Cingolani, P. , Platts, A. , Wang, L. L. , Coon, M. , Nguyen, T. , Wang, L. , … Ruden, D. M. (2012). A program for annotating and predicting the effects of single nucleotide polymorphisms, SnpEff. Fly (Austin), 6, 80–92. 22728672 10.4161/fly.19695 PMC3679285

[mec15120-bib-0020] Coffman, A. J. , Hsieh, P. H. , Gravel, S. , & Gutenkunst, R. N. (2016). Computationally efficient composite likelihood statistics for demographic inference. Molecular Biology and Evolution, 33, 591–593. 10.1093/molbev/msv255 26545922 PMC5854098

[mec15120-bib-0021] Cole, S. T. , Brosch, R. , Parkhill, J. , Garnier, T. , Churcher, C. , Harris, D. , … Barrell, B. G. (1998). Erratum: Deciphering the biology of *Mycobacterium tuberculosis* from the complete genome sequence. Nature, 396, 190 10.1038/24206 9634230

[mec15120-bib-0022] Comas, I. , Coscolla, M. , Luo, T. , Borrell, S. , Holt, K. E. , Kato‐Maeda, M. , … Gagneux, S. (2013). Out‐of‐Africa migration and Neolithic coexpansion of *Mycobacterium tuberculosis* with modern humans. Nature Genetics, 45, 1176–1182. 10.1038/ng.2744 23995134 PMC3800747

[mec15120-bib-0023] Comas, I. , Hailu, E. , Kiros, T. , Bekele, S. , Mekonnen, W. , Gumi, B. , … Berg, S. (2015). Population genomics of *Mycobacterium tuberculosis* in Ethiopia contradicts the virgin soil hypothesis for human tuberculosis in Sub‐Saharan Africa. Current Biology, 25, 3260–3266. 10.1016/j.cub.2015.10.061 26687624 PMC4691238

[mec15120-bib-0024] Coningham, R. , & Young, R. (Eds.) (2015). The archaeology of South Asia: From the Indus to Asoka, c.6500 BCE–200 CE. Cambridge, UK: Cambridge University Press.

[mec15120-bib-0025] de Jong, B. C. , Hill, P. C. , Aiken, A. , Awine, T. , Martin, A. , Adetifa, I. M. , … Adegbola, R. A. (2008). Progression to active tuberculosis, but not transmission, varies by *Mycobacterium tuberculosis* lineage in the Gambia. Journal of Infectious Diseases, 198, 1037–1043. 18702608 10.1086/591504 PMC2597014

[mec15120-bib-0026] De Maio, N. , Wu, C.‐H. , O'Reilly, K. M. , & Wilson, D. (2015). New routes to phylogeography: A Bayesian structured coalescent approximation. PLoS Genetics, 11, e1005421. 26267488 10.1371/journal.pgen.1005421 PMC4534465

[mec15120-bib-0027] De Mita, S. , & Siol, M. (2012). EggLib: Processing, analysis and simulation tools for population genetics and genomics. BMC Genetics, 13, 27. 22494792 10.1186/1471-2156-13-27 PMC3350404

[mec15120-bib-0028] Demay, C. , Liens, B. , Burguière, T. , Hill, V. , Couvin, D. , Millet, J. , … Rastogi, N. (2012). SITVITWEB – A publicly available international multimarker database for studying *Mycobacterium tuberculosis* genetic diversity and molecular epidemiology. Infection, Genetics and Evolution, 12, 755–766. 10.1016/j.meegid.2012.02.004 22365971

[mec15120-bib-0029] DePristo, M. A. , Banks, E. , Poplin, R. , Garimella, K. V. , Maguire, J. R. , Hartl, C. , … Daly, M. J. (2011). A framework for variation discovery and genotyping using next‐generation DNA sequencing data. Nature Genetics, 43, 491–498. 10.1038/ng.806 21478889 PMC3083463

[mec15120-bib-0030] Dilke, O. (1985). Greek and Roman maps. Baltimore, MD: Johns Hopkins University Press.

[mec15120-bib-0031] Dray, S. , & Dufour, A. B. (2007). The ade4 package: Implementing the duality diagram for ecologists. Journal of Statistical Software, 22, 3241–20.

[mec15120-bib-0032] Drummond, A. J. , & Rambaut, A. (2007). BEAST: Bayesian evolutionary analysis by sampling trees. BMC Evolutionary Biology, 7, 214. 17996036 10.1186/1471-2148-7-214 PMC2247476

[mec15120-bib-0033] Eldholm, V. , Pettersson, J.‐H.‐O. , Brynildsrud, O. B. , Kitchen, A. , Rasmussen, E. M. , Lillebaek, T. , … Balloux, F. (2016). Armed conflict and population displacement as drivers of the evolution and dispersal of *Mycobacterium tuberculosis* . Proceedings of the National Academy of Sciences of the United States of America, 113, 13881–13886. 27872285 10.1073/pnas.1611283113 PMC5137683

[mec15120-bib-0034] Erdkamp, P. (2002). The Roman army and the economy. Amsterdam, the Netherlands: Gieben.

[mec15120-bib-0035] Ewing, G. B. , & Jensen, J. D. (2015). The consequences of not accounting for background selection in demographic inference. Molecular Ecology, 25, 135–141. 10.1111/mec.13390 26394805

[mec15120-bib-0036] Firdessa, R. , Berg, S. , Hailu, E. , Schelling, E. , Gumi, B. , Erenso, G. , … Aseffa, A. (2013). Mycobacterial lineages causing pulmonary and extrapulmonary tuberculosis, Ethiopia. Emerging Infectious Diseases, 19, 460–463. 10.3201/eid1903.120256 23622814 PMC3647644

[mec15120-bib-0037] Gagneux, S. , DeRiemer, K. , Van, T. , Kato‐Maeda, M. , de Jong, B. C. , Narayanan, S. , … Small, P. M. (2006). Variable host–pathogen compatibility in *Mycobacterium tuberculosis* . Proceedings of the National Academy of Sciences of the United States of America, 103, 2869–2873. 10.1073/pnas.0511240103 16477032 PMC1413851

[mec15120-bib-0038] García‐Alcalde, F. , Okonechnikov, K. , Carbonell, J. , Cruz, L. M. , Götz, S. , Tarazona, S. , … Conesa, A. (2012). Qualimap: Evaluating next‐generation sequencing alignment data. Bioinformatics, 28, 2678–2679. 10.1093/bioinformatics/bts503 22914218

[mec15120-bib-0039] Government of Canada PHA of C . (2005). Tuberculosis prevention and control in Canada: A federal framework for action. Retrieved from http://www.phac-aspc.gc.ca/index-eng.php

[mec15120-bib-0040] Green, M. H. (2018). Climate and disease in Medieval Eurasia In Ludden D. (Ed.), Oxford research encyclopedia of Asian history. New York, NY: Oxford University Press 10.1093/acrefore/9780190277727.013.6

[mec15120-bib-0041] Gutenkunst, R. N. , Hernandez, R. D. , Williamson, S. H. , & Bustamante, C. D. (2009). Inferring the joint demographic history of multiple populations from multidimensional SNP frequency data. PLoS Genetics, 5, e1000695 10.1371/journal.pgen.1000695 19851460 PMC2760211

[mec15120-bib-0042] Hansen, V. (2012). The silk road: A new history. Oxford, UK: Oxford University Press.

[mec15120-bib-0043] Hernandez, R. D. (2008). A flexible forward simulator for populations subject to selection and demography. Bioinformatics, 24, 2786–2787. 10.1093/bioinformatics/btn522 18842601 PMC2639268

[mec15120-bib-0044] Hershberg, R. , Lipatov, M. , Small, P. M. , Sheffer, H. , Niemann, S. , Homolka, S. , … Gagneux, S. (2008). High functional diversity in *Mycobacterium tuberculosis* driven by genetic drift and human demography. PLoS Biology, 6, e311 10.1371/journal.pbio.0060311 19090620 PMC2602723

[mec15120-bib-0045] Hijmans, R. J. , Williams, E. , & Vennes, C. (2016). geosphere: Spherical trigonometry. Retrieved from https://cran.r-project.org/web/packages/geosphere/index.html

[mec15120-bib-0046] Hirsh, A. E. , Tsolaki, A. G. , DeRiemer, K. , Feldman, M. W. , & Small, P. M. (2004). Stable association between strains of *Mycobacterium tuberculosis* and their human host populations. Proceedings of the National Academy of Sciences of the United States of America, 101, 4871–4876. 15041743 10.1073/pnas.0305627101 PMC387341

[mec15120-bib-0047] Holt, K. E. , McAdam, P. , Thai, P. V. K. , Thuong, N. T. T. , Ha, D. T. M. , Lan, N. N. , … Dunstan, S. J. (2018). Frequent transmission of the *Mycobacterium tuberculosis* Beijing lineage and positive selection for the EsxW Beijing variant in Vietnam. Nature Genetics, 50, 849–856. 10.1038/s41588-018-0117-9 29785015 PMC6143168

[mec15120-bib-0048] Institute of Environmental Science and Research Limited . (2014). Tuberculosis in New Zealand: Annual report (2014). Retrieved from https://surv.esr.cri.nz/surveillance/AnnualTBReports.php?we_objectID=4251

[mec15120-bib-0049] Isaac, B. H. (2004). The limits of empire: The Roman army in the East. Oxford, UK: Clarendon Press.

[mec15120-bib-0050] Kamvar, Z. N. , Tabima, J. F. , & Grünwald, N. J. (2014). Poppr: An R package for genetic analysis of populations with clonal, partially clonal, and/or sexual reproduction. PeerJ, 2, e281. 24688859 10.7717/peerj.281 PMC3961149

[mec15120-bib-0051] Kay, G. L. , Sergeant, M. J. , Zhou, Z. , Chan, J.‐M. , Millard, A. , Quick, J. , … Pallen, M. J. (2015). Eighteenth‐century genomes show that mixed infections were common at time of peak tuberculosis in Europe. Nature Communications, 6, 6717 10.1038/ncomms7717 PMC4396363 25848958

[mec15120-bib-0052] Kent, R. K. (1979). The possibilities of Indonesian colonies in Africa with reference to Madagascar In Mouvements de Populations dans L'Ocean Indie (pp. 93–105). Paris, France: H. Champion.

[mec15120-bib-0053] Lapierre, M. , Blin, C. , Lambert, A. , Achaz, G. , & Rocha, E. P. C. (2016). The impact of selection, gene conversion, and biased sampling on the assessment of microbial demography. Molecular Biology and Evolution, 33, 1711–1725. 10.1093/molbev/msw048 26931140 PMC4915353

[mec15120-bib-0054] Leinonen, R. , Sugawara, H. , & Shumway, M. (2011). The sequence read archive. Nucleic Acids Research, 39, D19–D21. 10.1093/nar/gkq1019 21062823 PMC3013647

[mec15120-bib-0055] Lemey, P. , Rambaut, A. , Drummond, A. J. , & Suchard, M. A. (2009). Bayesian phylogeography finds its roots. PLoS Computational Biology, 5, e1000520 10.1371/journal.pcbi.1000520 19779555 PMC2740835

[mec15120-bib-0056] Li, H. (2013). Aligning sequence reads, clone sequences and assembly contigs with BWA‐MEM. ArXiv13033997 Q‐Bio [Internet]. Retrieved from http://arxiv.org/abs/1303.3997

[mec15120-bib-0057] Liu, Q. , Ma, A. , Wei, L. , Pang, Y. U. , Wu, B. , Luo, T. , … Gao, Q. (2018). China's tuberculosis epidemic stems from historical expansion of four strains of *Mycobacterium tuberculosis* . Nature Ecology and Evolution, 2, 1982 10.1038/s41559-018-0680-6 30397300 PMC6295914

[mec15120-bib-0058] Luo, T. , Comas, I. , Luo, D. , Lu, B. , Wu, J. , Wei, L. , … Gao, Q. (2015). Southern East Asian origin and coexpansion of *Mycobacterium tuberculosis* Beijing family with Han Chinese. Proceedings of the National Academy of Sciences of the United States of America, 112, 8136–8141. 26080405 10.1073/pnas.1424063112 PMC4491734

[mec15120-bib-0059] Luttwak, E. N. (1976). The grand strategy of the Roman Empire: From the first century A.D. to the third. London, UK: Weidenweld & Nicholson.

[mec15120-bib-0060] Martin, M. (2011). Cutadapt removes adapter sequences from high‐throughput sequencing reads. EMBnet.journal, 17, 10–12. 10.14806/ej.17.1.200

[mec15120-bib-0061] Menardo, F. , Duchene, S. , Brites, D. , & Gagneux, S. (2019). The molecular clock of *Mycobacterium tuberculosis* . bioRxiv. 10.1101/532390 PMC6759198 31513651

[mec15120-bib-0062] Millar, F. (1993). The Roman Near East, 31 B.C.‐A.D. 337. Cambridge, MA: Harvard University Press.

[mec15120-bib-0063] Mortimer, T. D. , Weber, A. M. , & Pepperell, C. S. (2018). Signatures of selection at drug resistance loci in *Mycobacterium tuberculosis* . mSystems, 3, e00108‐17 10.1128/mSystems.00108-17 29404424 PMC5790871

[mec15120-bib-0064] O'Neill, M. B. , Mortimer, T. D. , & Pepperell, C. S. (2015). Diversity of *Mycobacterium tuberculosis* across evolutionary scales. PLoS Pathology, 11, e1005257 10.1371/journal.ppat.1005257 PMC4642946 26562841

[mec15120-bib-0065] Oksanen, J. , Blanchet, F. G. , Friendly, M. , Kindt, R. , Legendre, P. , McGlinn, D. , Wagner, H. (2017). vegan: Community ecology package. Retrieved from https://cran.r-project.org/web/packages/vegan/index.html

[mec15120-bib-0066] Page, A. J. , Taylor, B. , Delaney, A. J. , Soares, J. , Seemann, T. , Keane, J. A. , & Harris, S. R. (2016). SNP‐sites: Rapid efficient extraction of SNPs from multi‐FASTA alignments. Retrieved from http://biorxiv.org/lookup/doi/10.1101/038190 10.1099/mgen.0.000056 PMC5320690 28348851

[mec15120-bib-0067] Paradis, E. , Claude, J. , & Strimmer, K. (2004). APE: Analyses of phylogenetics and evolution in R language. Bioinformatics, 20, 289–290. 10.1093/bioinformatics/btg412 14734327

[mec15120-bib-0068] Parkin, D. , & Barnes, R. (Eds.) (2002). Ships and the development of maritime technology in the Indian Ocean. London, UK: Routledge Curzon.

[mec15120-bib-0069] Pepperell, C. S. , Casto, A. M. , Kitchen, A. , Granka, J. M. , Cornejo, O. E. , Holmes, E. C. , … Feldman, M. W. (2013). The role of selection in shaping diversity of natural *M. tuberculosis* populations. PLoS Path, 9, e1003543. 10.1371/journal.ppat.1003543 PMC3744410 23966858

[mec15120-bib-0070] Pepperell, C. S. , Granka, J. M. , Alexander, D. C. , Behr, M. A. , Chui, L. , Gordon, J. , … Feldman, M. W. (2011). Dispersal of *Mycobacterium tuberculosis* via the Canadian fur trade. Proceedings of the National Academy of Sciences of the United States of America, 108, 6526–6531. 10.1073/pnas.1016708108 21464295 PMC3080970

[mec15120-bib-0071] Pfister, R. , & Bellinger, L. (1945). The excavations at Dura‐Europos. Final report IV: The Textiles. New Haven, CT: Yale University Press.

[mec15120-bib-0072] Price, M. N. , Dehal, P. S. , & Arkin, A. P. (2010). FastTree 2 – Approximately maximum‐likelihood trees for large alignments. PLoS ONE, 5, e9490 10.1371/journal.pone.0009490 20224823 PMC2835736

[mec15120-bib-0073] Public Health England . (2015). Tuberculosis in England: Annual report ‐ GOV.UK. Retrieved from https://www.gov.uk/government/publications/tuberculosis-in-england-annual-report

[mec15120-bib-0074] R Development Core Team . (2014). R: A language and environment for statistical computing. Vienna, Austria: R Foundation for Statistical Computing Retrieved from http://www.R-project.org/

[mec15120-bib-0075] Ramachandran, S. , Deshpande, O. , Roseman, C. C. , Rosenberg, N. A. , Feldman, M. W. , & Cavalli‐Sforza, L. L. (2005). Support from the relationship of genetic and geographic distance in human populations for a serial founder effect originating in Africa. Proceedings of the National Academy of Sciences of the United States of America, 102, 15942–15947. 10.1073/pnas.0507611102 16243969 PMC1276087

[mec15120-bib-0076] Rauh, N. K. (2003). Merchants, sailors and pirates in the Roman world. Stroud, UK: Tempus.

[mec15120-bib-0077] Ray, H. P. (Eds.) (2003). The archaeology of seafaring in ancient South Asia. Cambridge, UK: Cambridge University Press.

[mec15120-bib-0078] Ray, H. P. , & Salles, J.‐F. (1996). Institute of Southeast Asian Studies, Maison de l'Orient méditerranéen ancien (Lyon F, National Institute of Science, Technology and Development Studies, France, Ambassade (India), Centre for Human Sciences Tradition and archaeology: Early maritime contacts in the Indian Ocean. New Delhi, India: Manohar Publishers.

[mec15120-bib-0079] Sabin, S. , Herbig, A. , Vågene, Å. J. , Ahlström, T. , Bozovic, G. , Arcini, C. , … Bos, K. I. (2019). A seventeenth‐century *Mycobacterium tuberculosis* genome supports a Neolithic emergence of the *Mycobacterium tuberculosis* complex. bioRxiv. 10.1101/588277 PMC7418204 32778135

[mec15120-bib-0080] Salles, J.‐F. (1996). Achaemenid and Hellenistic trade in the Indian Ocean The Indian Ocean in antiquity (pp. 251–267). Retrieved from http://public.eblib.com/choice/publicfullrecord.aspx?p=1517609

[mec15120-bib-0081] Sartre, M. (1991). L'Orient romain: Provinces et sociétés provinciales en Méditerranée orientale d'Auguste aux Sévères (31 avant J.‐C‐235 après J.‐C.). Paris, France: Seuil.

[mec15120-bib-0082] Shabbeer, A. , Cowan, L. S. , Ozcaglar, C. , Rastogi, N. , Vandenberg, S. L. , Yener, B. , & Bennett, K. P. (2012). TB‐Lineage: An online tool for classification and analysis of strains of *Mycobacterium tuberculosis* complex. Infection Genetics and Evolution, 12, 789–797. 10.1016/j.meegid.2012.02.010 22406225

[mec15120-bib-0083] Shannon, P. , Markiel, A. , Ozier, O. , Baliga, N. S. , Wang, J. T. , Ramage, D. , … Ideker, T. (2003). Cytoscape: A software environment for integrated models of biomolecular interaction networks. Genome Research, 13, 2498–2504. 10.1101/gr.1239303 14597658 PMC403769

[mec15120-bib-0084] South, A. (2016). rworldmap: Mapping global data. Retrieved from https://cran.r-project.org/web/packages/rworldmap/index.html

[mec15120-bib-0085] Stamatakis, A. (2014). RAxML version 8: A tool for phylogenetic analysis and post‐analysis of large phylogenies. Bioinformatics, 30, 1312–1313. 10.1093/bioinformatics/btu033 24451623 PMC3998144

[mec15120-bib-0086] Thorpe, H. A. , Bayliss, S. C. , Hurst, L. D. , & Feil, E. J. (2017). Comparative analyses of selection operating on nontranslated intergenic regions of diverse bacterial species. Genetics, 206, 363–376. 10.1534/genetics.116.195784 28280056 PMC5419481

[mec15120-bib-0087] Vogt, B. (1996). Bronze Age maritime trade in the Indian Ocean: Harappan traits on the Oman Peninsula In Reade J. (Ed.), The Indian Ocean in antiquity (pp. 107–132). London, UK: Kegan Paul International in association with the British Museum Retrieved from http://public.eblib.com/choice/publicfullrecord.aspx?p=1517609

[mec15120-bib-0088] Walker, B. J. , Abeel, T. , Shea, T. , Priest, M. , Abouelliel, A. , Sakthikumar, S. , … Earl, A. M. (2014). Pilon: An integrated tool for comprehensive microbial variant detection and genome assembly improvement. PLoS ONE, 9, e112963 10.1371/journal.pone.0112963 25409509 PMC4237348

[mec15120-bib-0089] White, Z. , Painter, J. , Douglas, P. , Abubakar, I. , Njoo, H. , Archibald, C. , … Posey, D. L. (2017). Immigrant arrival and tuberculosis among large immigrant‐ and refugee‐receiving countries, 2005–2009. Tuberculosis Research and Treatment, 2017, 10.1155/2017/8567893 PMC5382300 28424748

[mec15120-bib-0090] Wink, A. (2002). From the mediterranean to the Indian Ocean: Medieval history in geographic perspective. Comparative Studies in Society and History, 44, 416–445. 10.1017/S001041750200021X

[mec15120-bib-0091] World Health Organization . (2017). Global tuberculosis report 2017. World Health Organization.

[mec15120-bib-0092] Yu, G. , Smith, D. K. , Zhu, H. , Guan, Y. , & Lam, T.‐T.‐Y. (2017). ggtree: An r package for visualization and annotation of phylogenetic trees with their covariates and other associated data. Methods in Ecology and Evolution, 8, 28–36.

[mec15120-bib-0093] Zarins, J. (1996). Obsidian in the larger context of Predynastic/Archaic Egyptian Red Sea trade In Reade J. (Ed.), The Indian Ocean in Antiquity (pp. 107–132). London, UK: Kegan Paul International in association with the British Museum.

