# Supplementary figures and images for: Lineage specific histories of Mycobacterium tuberculosis dispersal in Africa and Eurasia
Source: Mol Ecol. 2019 Jul 9;28(13):3241–56. doi: 10.1111/mec.15120 (PMC6660993; doi:10.1111/mec.15120)

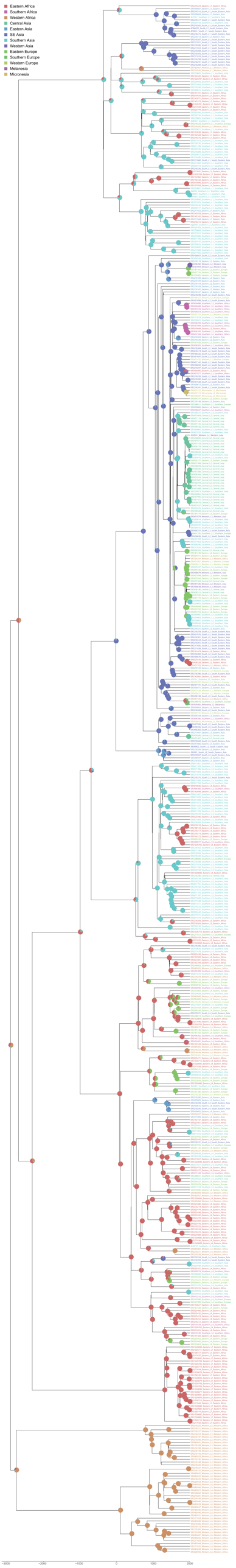

Supplement: Supplementary file 2 [file MEC-28-3241-s002.pdf]
